# Nonlinear Dynamics and Entropy of Complex Systems with Hidden and Self-Excited Attractors II

**DOI:** 10.3390/e22121428

**Published:** 2020-12-18

**Authors:** Christos K. Volos, Sajad Jafari, Jesus M. Munoz-Pacheco, Jacques Kengne, Karthikeyan Rajagopal

**Affiliations:** 1 Laboratory of Nonlinear Systems, Circuits & Complexity (LaNSCom), Department of Physics, Aristotle University of Thessaloniki, Thessaloniki 54124, Greece; 2 Nonlinear Systems and Applications, Faculty of Electrical and Electronics Engineering, Ton Duc Thang University, Ho Chi Minh City 700000, Vietnam; sajad.jafari@tdtu.edu.vn; 3 Faculty of Electronics Sciences, Autonomous University of Puebla, Puebla 72000, Mexico; jesusm.pacheco@correo.buap.mx; 4 Department of Electrical Engineering, University of Dschang, P.O. Box 134 Dschang, Cameroon; kengnemozart@yahoo.fr; 5 Center for Nonlinear Systems, Chennai Institute of Technology, Chennai 600069, India; rkarthiekeyan@gmail.com or

According to the pioneering work of Leonov and Kuznetsov [1], the attractors in complex systems can be classified as self-excited and hidden attractors. While a self-excited attractor presents a basin of attraction associated with an unstable equilibrium, a hidden attractor has a basin of attraction that does not intersect with small neighborhoods of the unstable equilibrium. Hence, the paradigm of deterministic chaos for complex nonlinear systems is becoming increasingly popular for its attractive concept and successful real-world applications. 

In this framework, it is essential to study the complexity of nonlinear systems. Nonlinear complexity measures may include fractal dimensions, correlation dimension, Lyapunov exponents, K-S entropy, approximate entropy, permutation entropy, and multiscale entropy. Additionally, in complex systems with hidden and self-excited attractors, several behaviors, such as multistability, extreme multistability, coexisting attractors, and transient chaos, can be observed as functions of the system’s parameters and initial conditions. By introducing a fractional order for the derivatives of dynamical systems, the memory properties can be also considered. There is evidence that the mentioned dynamics play a vital role in various fields ranging from living systems (economics, neural networks, neural computing, neurostimulation, and so on) to nonliving systems (control theory, circuits, memristors, encryption, random number generators, etc.).

The overall purpose of the second volume of this Special Issue is to gather the latest scientific advances on topics of dynamics, entropy, fractional-order calculus, and applications in complex systems with hidden attractors and self-excited attractors.

In the paper “A Giga-Stable Oscillator with Hidden and Self-Excited Attractors: A Megastable Oscillator Forced by His Twin”, Thoai Phu Vo, Yeganeh Shaverdi, Abdul Jalil M. Khalaf, Fawaz E. Alsaadi, Tasawar Hayat, and Viet-Thanh Pham proposed a modified nonlinear oscillator which has an infinite number of coexisting torus attractors, strange attractors, and limit cycle attractors. By calculating the entropy, energy, and homogeneity of attractors’ images and basins of attraction, they showed an increase in the complexity of attractors when changing the bifurcation parameters [2].

In the paper “A Class of Quadratic Polynomial Chaotic Maps and Their Fixed Points Analysis”, Chuanfu Wang and Qun Ding studied a class of quadratic polynomial chaotic maps. They found that the polynomial chaotic maps satisfy the Li–Yorke definition of chaos, and through the existence and stability analysis of fixed points, they proved that such class quadratic polynomial maps cannot have hidden chaotic attractors [3].

In the paper “Coexisting Attractors and Multistability in a Simple Memristive Wien-Bridge Chaotic Circuit”, Yixuan Song, Fang Yuan, and Yuxia Li presented a new absolute voltage-controlled memristor and described the construction of a simple three-order Wien-bridge chaotic circuit without inductor on the basis of the proposed memristor. They observed coexisting attractors and multistability that could be used to obtain a pseudorandom sequence generator for digital encryption systems. Therefore, the chaotic system was discretized and implemented by Digital signal processing (DSP) technology, and the National Institute of Standards and Technology (NIST) and approximate entropy analyses were computed [4].

In the paper “Image Encryption Scheme with Compressed Sensing Based on New Three-Dimensional Chaotic System”, Yaqin Xie, Jiayin Yu, Shiyu Guo, Qun Ding, and Erfu Wang proposed a new three-dimensional chaotic system for image encryption. The performance analysis showed that the chaotic system has two positive Lyapunov exponents and high complexity. In the encryption scheme, the new chaotic system was used as the measurement matrix for compressed sensing, and Arnold was used for scrambling the image further [5].

In the paper “New Nonlinear Active Element Dedicated to Modeling Chaotic Dynamics with Complex Polynomial Vector Fields”, Jiri Petrzela and Roman Sotner described the evolution of a new active element that is able to significantly simplify the design process of a lumped chaotic oscillator. The major advantage of the proposed active device is the incorporation of two fundamental mathematical operations into a single five-port voltage-input current-output element. The developed active device was verified by three different synthesis scenarios [6].

In the paper “Entropy Analysis and Image Encryption Application Based on a New Chaotic System Crossing a Cylinder”, Alaa Kadhim Farhan, Nadia M.G. Al-Saidi, Abeer Tariq Maolood, Fahimeh Nazarimehr, and Iqtadar Hussain presented a novel chaotic system with a unique feature of crossing inside and outside of a cylinder repeatedly, as well as multistability. The system was characterized using the bifurcation diagram, Lyapunov exponents’ spectrum, and entropy measurement. Due to the complexity of the chaotic attractor, it was applied as the core of an encryption method [7].

In the paper “A High Spectral Entropy (SE) Memristive Hidden Chaotic System with Multi-Type Quasi-Periodic and its Circuit”, Licai Liu, Chuanhong Du, Lixiu Liang, and Xiefu Zhang proposed a novel five-dimensional chaotic system with high complexity and hidden attractors based on a flux-controlled memristor. The proposed system had a very high complexity when measured through spectral entropy. The authors also found multistability and transient chaos phenomena. Finally, a physical realization by means of electronic components was presented [8].

In the paper “Low-Element Image Restoration Based on an Out-of-Order Elimination Algorithm”, Yaqin Xie, Jiayin Yu, Xinwu Chen, Qun Ding, and Erfu Wang introduced an image restoration method for underdetermined blind-source separation based on an out-of-order elimination algorithm with the aim of realizing fewer devices at the receiving end without information loss. The results showed that the performance of the underdetermined blind separation algorithm is related to the configuration of the transceiver antenna [9].

In the paper “Image Parallel Encryption Technology Based on Sequence Generator and Chaotic Measurement Matrix”, Jiayin Yu, Shiyu Guo, Xiaomeng Song, Yaqin Xie, and Erfu Wang reported a new image encryption transmission algorithm based on the parallel mode. To improve efficiency, the paper adopted the method of parallel compressed sensing to realize image transmission. Simulation experiments and analyses showed that the algorithm has the capacity to resist illegal attacks [10].

In the paper “Stabilization of Port Hamiltonian Chaotic Systems with Hidden Attractors by Adaptive Terminal Sliding Mode Control”, Ahmad Taher Azar and Fernando E. Serrano designed an adaptive terminal sliding mode controller for the stabilization of port Hamiltonian chaotic systems with hidden attractors. A Lyapunov approach was used to formulate the adaptive device controller by creating a control law and an adaptive law, which were used online to make the system states stable while at the same time suppressing their chaotic behavior [11].

In the paper “Dynamic Effects Arise Due to Consumers’ Preferences Depending on Past Choices”, Sameh S. Askar and A. Al-khedhairi analyzed a dynamic duopoly game where players adopt specific preferences. The authors investigated two possible cases for the suggested game. For the second case, a numerical simulation was carried out to perform local and global investigations of the chaotic behavior of the game’s map. In addition, they computed the entropy to obtain more information about the regularity and predictability of the time series [12].

In the paper “Synchronization of a Non-Equilibrium Four-Dimensional Chaotic System Using a Disturbance-Observer-Based Adaptive Terminal Sliding Mode Control Method”, Shaojie Wang, Amin Yousefpour, Abdullahi Yusuf, Hadi Jahanshahi, Raúl Alcaraz, Shaobo He, and Jesus M. Munoz-Pacheco studied the dynamical behavior and synchronization of a nonequilibrium four-dimensional chaotic system. The authors proposed a new disturbance-observer-based adaptive terminal sliding mode control (ATSMC) method with input saturation for the control and synchronization of the chaotic system. Numerical simulations were presented to demonstrate the performance of the designed control scheme in the presence of noise, disturbances, and control input saturation [13].

In the paper “Investigation of Early Warning Indexes in a Three-Dimensional Chaotic System with Zero Eigenvalues”, Lianyu Chen, Fahimeh Nazarimehr, Sajad Jafari, Esteban Tlelo-Cuautle, and Iqtadar Hussain reported a rare three-dimensional chaotic system with all eigenvalues equal to zero. The authors observed that from the evaluation of the entropy (Shannon and Kolmogorov–Sinai entropy), bifurcation points can be predicted by identifying early warning signals [14].

In the paper “Fractional-Order Chaotic Memory with Wideband Constant Phase Elements”, Jiri Petrzela studied three cases. Firstly, the gallery of the so-called constant phase elements (CPEs) designed for wideband applications, was presented. Secondly, the dynamics of ternary memory composed of a series connection of two resonant tunneling diodes was investigated. Finally, CPEs were directly used for the realization of fractional-order (FO) ternary memory as a lumped chaotic oscillator. By numerical and experimental measurements, the strange attractors for different orders were demonstrated [15].

In the paper “Modification of the Logistic Map Using Fuzzy Numbers with Application to Pseudorandom Number Generation and Image Encryption”, Lazaros Moysis, Christos Volos, Sajad Jafari, Jesus M. Munoz-Pacheco, Jacques Kengne, Karthikeyan Rajagopal, and Ioannis Stouboulos proposed a modification of the classic logistic map using fuzzy triangular numbers. It showed higher complexity compared to the classic logistic map and showcased phenomena such as antimonotonicity and crisis. The map was then applied to the problem of pseudorandom bit generation, using a simple rule to generate the bit sequence [16].

In the paper “Neural Computing Enhanced Parameter Estimation for Multi-Input and Multi-Output Total Non-Linear Dynamic Models”, Longlong Liu, Di Ma, Ahmad Taher Azar, and Quanmin Zhu reported a gradient descent algorithm for the parameter estimation of multi-input and multioutput (MIMO) total nonlinear dynamic models. Firstly, the MIMO total nonlinear model was mapped to a noncompletely connected feedforward neural network. The authors proposed a weight-updating algorithm with the convergence conditions of a noncompletely connected feedforward network. Moreover, they introduced a method of model structure detection for selecting a group of important items from the whole variable candidate set [17].

In the paper “Birhythmic Analog Circuit Maze: A Nonlinear Neurostimulation Testbed”, Ian D. Jordan and II Memming Park designed an analog circuit as a model for the multistable brain dynamics. They observed that the circuit spontaneously oscillates stably on two periods as an instantiation of a three-dimensional continuous-time gated recurrent neural network. They demonstrated the existence of nontrivial solutions to the transition problem in the circuit implementation [18].

In the paper “Chaos Control and Synchronization of a Complex Rikitake Dynamo Model”, Wenkai Pang, Zekang Wu, Yu Xiao, and Cuimei Jiang proposed a novel chaotic system called the complex Rikitake system. Dynamical properties, including symmetry, dissipation, stability of equilibria, Lyapunov exponents, and bifurcation, were analyzed on the basis of theoretical analysis and numerical simulation. Additionally, the authors not only proved the existence of two types of synchronization schemes in the complex Rikitake system, but also designed adaptive controllers [19].

The guest editors hope you will delight in reading the second volume of this Special Issue focused on cutting-edge research on nonlinear dynamics, entropy, and chaos. We expect the collected works will motivate researchers to strive for further advances in the emerging areas of complex systems with hidden and self-excited attractors in both integer and fractional-order domains.
